# Particulate matter exposure during pregnancy is associated with birth weight, but not gestational age, 1962-1992: a cohort study

**DOI:** 10.1186/1476-069X-11-13

**Published:** 2012-03-09

**Authors:** Mark S Pearce, Svetlana V Glinianaia, Rakesh Ghosh, Judith Rankin, Steven Rushton, Martin Charlton, Louise Parker, Tanja Pless-Mulloli

**Affiliations:** 1 Institute of Health and Society, Newcastle University, Newcastle upon Tyne, England, UK; 2 Department of Public Health Sciences, University of California, Davis, California, USA; 3 Newcastle Institute for Research on Sustainability, Newcastle University, Newcastle upon Tyne, England, UK; 4 National Centre for Geocomputation, National University of Ireland, Maynooth, Ireland; 5 Departments of Medicine and Pediatrics, Dalhousie University, Halifax, Nova Scotia, Canada

**Keywords:** Black smoke, Particulate matter, Air pollution, Birth weight, Gestational age

## Abstract

**Background:**

Exposure to air pollutants is suggested to adversely affect fetal growth, but the evidence remains inconsistent in relation to specific outcomes and exposure windows.

**Methods:**

Using birth records from the two major maternity hospitals in Newcastle upon Tyne in northern England between 1961 and 1992, we constructed a database of all births to mothers resident within the city. Weekly black smoke exposure levels from routine data recorded at 20 air pollution monitoring stations were obtained and individual exposures were estimated via a two-stage modeling strategy, incorporating temporally and spatially varying covariates. Regression analyses, including 88,679 births, assessed potential associations between exposure to black smoke and birth weight, gestational age and birth weight standardized for gestational age and sex.

**Results:**

Significant associations were seen between black smoke and both standardized and unstandardized birth weight, but not for gestational age when adjusted for potential confounders. Not all associations were linear. For an increase in whole pregnancy black smoke exposure, from the 1^st ^(7.4 μg/m^3^) to the 25^th ^(17.2 μg/m^3^), 50^th ^(33.8 μg/m^3^), 75^th ^(108.3 μg/m^3^), and 90^th ^(180.8 μg/m^3^) percentiles, the adjusted estimated decreases in birth weight were 33 g (SE 1.05), 62 g (1.63), 98 g (2.26) and 109 g (2.44) respectively. A significant interaction was observed between socio-economic deprivation and black smoke on both standardized and unstandardized birth weight with increasing effects of black smoke in reducing birth weight seen with increasing socio-economic disadvantage.

**Conclusions:**

The findings of this study progress the hypothesis that the association between black smoke and birth weight may be mediated through intrauterine growth restriction. The associations between black smoke and birth weight were of the same order of magnitude as those reported for passive smoking. These findings add to the growing evidence of the harmful effects of air pollution on birth outcomes.

## Background

Evidence on the potential adverse impact of ambient air pollution on the health of adults and children has grown rapidly over the last two decades. It is now established that short and long-term increases in ambient air pollution are associated with increased mortality and morbidity in adults and children [1-4]. A growing body of evidence also suggests that exposure to ambient air pollutants, including particulate matter, adversely affects the growth and development of the fetus, such as growth restriction, preterm birth and congenital anomaly [5-12] and fetal and infant survival [13-15]. A systematic review of the literature summarising the literature on the association between maternal exposure to particulate matter and some fetal outcomes concluded that the currently available evidence was consistent with a small adverse effect of particulate air pollution on fetal growth and duration of pregnancy [16]. However, this and other reviews have emphasized the substantial inconsistency in the methods of the published studies, with confounding and exposure misclassification being identified as limitations of particular concern [7-18]. Few studies to date have used individual estimation of maternal exposure to air pollutants during pregnancy with many having to rely on routinely monitored data for mothers residing within a certain distance/radius from the monitor, as a proxy for exposure estimation. This effectively treats the monitoring station as a point source for exposure, rather than taking into account additional information such as industrial land-use and both industrial and residential chimneys, or when taking an ecological approach provides much uncertainty regarding individual-level exposures.

The aim of this study was to investigate the potential associations between individual weekly black smoke exposure estimates and birth weight, gestational age and birth weight standardized for gestational age and sex, in an urban setting. We used data from the United Kingdom (UK) Particulate Matter and Perinatal Events Research (PAMPER) study, set in a single conurbation in northern England (1962-1992) with a largely stable population. The PAMPER study is one of only a few to estimate individual-level exposure to particulate matter during pregnancy using a combination of monitored levels of black smoke (i.e. particulate matter with an aerodynamic diameter of 4 micron or less) and detailed pollution source information.

## Methods

The PAMPER birth cohort consists of all singletons born during 1961-1992 to mothers resident in the city of Newcastle upon Tyne in the north of England [19,20]. The population structure of the northern region of England is characterized by a low percentage of ethnic minorities (around 2%) and a relatively stable population with low levels of both inward and outward migration [21-23].

An electronic database of birth records was constructed using data from several sources. The main source was paper-based neonatal records from the two major maternity hospitals in Newcastle: The Princess Mary Maternity Hospital, where neonatal records were available for the entire study period, and Newcastle General Hospital, where records were available from 1967 onwards. Neonatal records from both hospitals contained data on important maternal and fetal/infant characteristics, including maternal name, home address at delivery, baby's name, sex, date and time of birth, gestational age, birth weight and survival status at birth. Data from 'birth ledgers', which are essentially lists of all births whether born in hospital or not, but also containing information on mother's current surname and residential address and the sex, date and place of birth and vital status of the baby, covering 1961-1973 were also included. Additional birth records from Newcastle General Hospital were accessed to complete some key variables which were not in the birth ledgers, including birth weight and gestational age. Home births, except those for the few where the mother was admitted to a maternity hospital shortly after delivery, were only recorded on the birth ledgers and hence had missing birth weight and gestational age information. Birth weights were standardized for gestational age and sex by calculating the z-score of birth weight for gestation separately for males and females using local, northern England, birth weight for gestational age standards [24]. This results in a standard deviation score based on the difference in birth weight from the mean in the reference population for each gestational age and sex group.

All births were assigned spatial identifiers (zipcodes and/or grid references). For births prior to 1970 (when zipcodes were introduced in the UK), the address at birth was assigned a zipcode according to those in place in 1991 and converted to a grid reference (equivalent to X-Y co-ordinates). Where zipcodes could not be completely identified, a grid reference was allocated directly. Grid references allowed linkage with UK census data. Through this, Townsend deprivation scores, an area-based measure of socio-economic status, incorporating home and car ownership, unemployment and overcrowding [25], were calculated at the enumeration district level (corresponding to around 450 people in 200 households) using data from the 1971, 1981 and 1991 census surveys.

### Estimating individual weekly exposure to black smoke

Black smoke is a historic measure of airborne particulate matter. Daily black smoke levels, with black smoke approximately equivalent to PM_4_, were obtained from routine data recorded at 20 air pollution monitoring stations within the city boundary between October 1961 and December 1992 and available from the UK Air Quality Archive. The city boundary used was based on the zipcodes NE1-NE7 and part of NE15, excluding Throckley, with the River Tyne forming the Southern boundary. An exact map of the study area is available in a previous publication [19]. Historical records were used to identify industrial land use and numbers of industrial and residential chimneys. The primary sources of air pollution in the study area, for the earlier part of the study were industrial and domestic coal combustion. As this decreased over the study period, the air pollution from road traffic increased and is now likely the largest source of air pollution in the area. Over the whole study period, the number of active monitors varied during any given week between three and ten. Black smoke levels for each individual birth were estimated using a combination of air pollution data, date of birth, estimated date of conception (based on date of last menstrual period) and the mother's residential zipcode which identified the location at which black smoke levels were to be estimated.

The modeling process to estimate individual exposure estimates is described in detail elsewhere [26]. Briefly, a two-stage modeling strategy was used. First, a seasonally varying temporal trend in black smoke exposures was estimated using a dynamic linear model. Secondly, the remaining spatio-temporal variation was accounted for using temporal and/or spatial covariates (the number of chimneys within 500 m of a monitor, distance of monitor to nearest industry, type of land use and implementation of the Clean Air Act). The residual spatio-temporal correlation remaining after this process was negligible. The two-stage exposure model used for individual exposure estimation explained 84% of the variation in black smoke levels at the locations of the monitoring stations. Mean weekly exposures were estimated for each birth, averaged for the whole pregnancy and for each trimester. As black smoke data were only available from 1962, only births with complete exposure estimates for the whole of pregnancy were included.

### Statistical analysis

Linear regression was used to examine potential associations between black smoke and the three outcome variables (birth weight, gestational age and standardized birth weight), adjusting for year of birth, maternal age, parity, Townsend deprivation score, sex and in the case of birth weight, gestational age. Birth weight and gestational age were treated as continuous variables, maternal age (in five-year groups), parity and sex were treated as categorical. From these models, coefficients per 10 μg/m^3 ^are given with corresponding 95% confidence intervals (95% CI). Additionally, if there was evidence of non-linear associations, an alternative functional form was established using fractional polynomials [27]. For such models, the associations are reported at a range of percentiles of exposure (1^st^, 25^th^, 50^th^, 75^th ^and 90^th^). Interactions between variables were assessed within the linear regression modelling framework. *P*-values are two-sided. The statistical software package STATA, version 9, (StataCorp: College Station, TX) was used for all analyses.

The study received a favorable ethical opinion (akin to approval) from the Sunderland Local Research Ethics Committee (SL REC 1071).

## Results

A total of 109,086 singleton births to mothers resident in Newcastle for the period 1961-1992 were identified [19]. Complete covariate information for the birth weight, standardized birth weight and gestational age models was available for 88,679 (81.3%), 87,412 (80.1%) and 88,863 (81.5%) births respectively. Births with missing covariate information were mostly home births during the early part of the study period (early 1960s) for whom birth weight and/or gestational age were not available [19].

Table 1 shows mean (SD) birth weight and gestational age by Townsend deprivation score, maternal age, parity and sex. For all births, mean birth weight increased and mean gestational age decreased during the study period. Mean birth weight significantly increased from 3,227 g (SD 661) in 1961 to 3,307 g (SD 572) in 1992. Mean gestational age decreased from 39.5 (SD 2.4) to 39.0 (SD 2.0) weeks during the study period, equivalent to four days.

**Table 1:** Mean (SD) of Birth Weight and Gestational age by Social and Demographic Characteristics for births in Newcastle upon Tyne, 1962-1992

Variables	Number of births with birth weight (gestational age)	Mean birth weight (SD) [grams]	Mean gestational age (SD) [weeks]
**Townsend deprivation score**			

1^st ^quintile (most advantaged)	18,943 (18,708)	3,363 (533)	39.4 (5.0)

2^nd ^quintile	18,805 (18,510)	3,305 (564)	39.4 (6.0)

3^rd ^quintile	18,813 (18,408)	3,271 (563)	39.4 (6.6)

4^th ^quintile	18,820 (18,252)	3,232 (572)	39.3 (7.6)

5^th ^quintile (most disadvantaged)	18,848 (18,200)	3,194 (580)	39.1 (8.9)

**Maternal age (years)**			

≤ 19	12,417 (11,868)	3,179 (553)	39.2 (2.2)

20-24	31,358 (30,519)	3,249 (552)	39.4 (2.1)

25-29	29,225 (28,766)	3,305 (555)	39.4 (2.0)

30-34	15,534 (15,225)	3,334 (573)	39.3 (2.0)

35-39	5,724 (5,608)	3,289 (639)	39.1 (2.1)

≥ 40	1,431 (1,384)	3,246 (655)	39.0 (2.2)

**Sex**			

Male	49,815 (48,688)	3,332 (579)	39.3 (2.1)

Female	46,627 (45,554)	3,210 (545)	39.3 (2.0)

**Parity**			

0 (Primipara)	42,179 (41,216)	3,228 (546)	39.4 (2.1)

1	28,635 (28,033)	3,324 (549)	39.3 (1.9)

2	13,674 (13,368)	3,310 (575)	39.2 (2.2)

3	5,672 (5,534)	3,278 (621)	39.2 (2.2)

≥ 4	6,083 (5,946)	3,283 (644)	39.3 (2.3)

Table 2 summarizes the average weekly exposure estimates over the whole pregnancy period and for each trimester. City-wide black smoke levels declined from above 500 μg/m^3 ^in the 1960s to around 20 μg/m^3 ^in the 1990. Black smoke exposures and year of birth were highly correlated (r = -0.84 for the whole pregnancy period, for example). The seasonal variation of black smoke persisted throughout the study period [26]. The variability of exposure estimates decreased in line with declining ambient black smoke levels [26]. All individual weekly exposure estimates above 200 μ/gm^3 ^occurred before 1970 and all below 50 μ/gm^3 ^after 1968.

**Table 2:** Summary statistics for average weekly black smoke exposure estimates (μg/m^3^) averaged by gestational period, for births in Newcastle upon Tyne 1962-92

Exposure window	Minimum	Median	Maximum	IQR
Whole pregnancy	3.7	**33.8**	499	**17-108**

Trimester 1	3.3	**32.4**	749	**17-94**

Trimester 2	3.1	**32.0**	722	**17-95**

Trimester 3	3.1	**30.8**	714	**16-95**

**Note**: *IQR *inter-quartile range

There were significant inverse linear associations between weekly black smoke exposure and all three outcomes when using the average weekly exposures across the whole of pregnancy, which remained for both birth weight variables following adjustment for other covariates, but not for gestational age (Table 3). Similar associations were seen for each trimester, with a trend towards increasingly strong associations with later trimesters, although, in the adjusted models, significant associations were not seen for either birth weight variable in the first trimester or for standardized birth weight in the third trimester.

**Table 3:** Unadjusted and adjusted linear regression results for black smoke (per 10 gm^3^) in relation to all three outcome measures, by exposure window

	Unadjusted		Adjusted*	
	**Coeff (95% CI)**	***P***	**Coeff (95% CI)**	***P***

**Whole pregnancy**				

Birth weight (g)	-3.4 (-3.9, -2.9)	< 0.00001	-1.7 (-2.4 -0.9)	< 0.00001

Gestational age (wks)	0.02 (0.01, 0.02)	< 0.0001	0.001 (-0.003, 0.004)	0.667

Standardized birth weight	-0.01 (-0.01; -0.01**)**	< 0.0001	-0.002 (-0.004 -0.0003)	0.020

**Trimester 1**				

Birth weight (g)	-0.2 (-2.4, -1.6)	< 0.0001	-0.2 (-0.7, 0.3)	0.497

Gestational age (wks)	0.01 (0.01, 0.02)	< 0.0001	0.004 (0.002, 0.006)	< 0.0001

Standardized birth weight	-0.007 (-0.008 -0.006)	< 0.0001	0.0003 (-0.0009; 0.001)	0.657

**Trimester 2**				

Birth weight (g)	-2.7 (-3.1, -2.3)	< 0.0001	-1.5 (-2.0, -0.9)	< 0.0001

Gestational age (wks)	0.01 (0.01, 0.02)	< 0.0001	0.003 (0.001, 0.006)	0.006

Standardized birth weight	-0.008(-0.009 -0.007)	< 0.0001	-0.002 (003; -0.001)	< 0.0001

**Trimester 3**				

Birth weight (g)	-3.0 (-3.4, -2.5)	< 0.0001	-0.7 (-1.2, -0.2)	0.012

Gestational age (wks)	0.01 (0.01, 0.02)	< 0.0001	-0.01(-0.01, -0.003)	< 0.0001

Standardized birth weight	-0.008(-0.008; -0.007)	< 0.0001	-0.0008 (-0.002, 0.0004)	0.203

*Adjusted for year of birth, maternal age, parity, Townsend deprivation score, sex and in the case of birth weight, additionally for gestational age. Sex and gestational age were not included as adjustment variables for standardized birth weight was they were part of the standardization process.

The associations between black smoke and unstandardized birth weight were significantly non-linear for whole pregnancy and each trimester. For whole pregnancy, the best fitting fractional polynomial model was β_1_*x*^*-*2^+ β_2_*x*^-0.5^, and for the first, second and third trimesters, the best fitting functional forms were *x*^*-*1^, ln(*x*) and *x*^-0.5^, respectively, where *x *denotes weekly average black smoke averaged over the entire duration of pregnancy or the corresponding trimester. For an increase in whole pregnancy exposure, from the 1^st ^(7.4 μg/m^3^) to the 25^th ^(17.2 μg/m^3^), 50^th ^(33.8 μg/m^3^), 75^th ^(108.3 μg/m^3^), and 90^th ^(180.8 μg/m^3^) percentiles the adjusted estimated decreases in birth weight were 33 g (SE 1.05), 62 g (1.63), 98 g (2.26) and 109 g (2.44) respectively. For an increase in trimester 1 exposure, from the 1^st ^percentile (6.7 μg/m^3^) to the 25^th ^(16.5 μg/m^3^), 50^th ^(32.4 μg/m^3^), 75^th ^(94.1 μg/m^3^) and 90^th ^(189.5 μg/m^3^) percentiles, the adjusted estimated decreases in birth weight were 21 g (SE 0.68), 28 g (0.91), 32 g (1.06) and 33 g (1.10) respectively.

For an increase in trimester 2 exposure, from the 1^st ^percentile (6.3 μg/m^3^) to the 25^th ^(16.5 μg/m^3^), 50^th ^(31.64 μg/m^3^), 75^th ^(94.8 μg/m^3^) and 90^th ^(186.7 μg/m^3^) percentiles, the adjusted estimated decreases in birth weight were 17 g (3.15), 28 g (5.28), 48 g (8.87) and 60 g (11.1) respectively.

For an increase in trimester 3 exposure, from the 1^st ^percentile (6.1 μg/m^3^) to the 25^th ^(15.9 μg/m^3^), 50^th ^(30.1 μg/m^3^), 75^th ^(91.0 μg/m^3^) and 90^th ^(182.4 μg/m^3^) percentiles, the adjusted estimated decreases in birth weight were 25 g (SE 1.77), 36 g (2.56), 49 g (3.46) and 54 g (3.82) respectively.

For standardized birth weight, the log of exposure β × log(x) was the best fitting fractional polynomial form for the whole pregnancy exposure, while linear models were used for exposures by trimester, because there was no evidence of non-linear associations. For an increase in whole pregnancy exposure, from the 1^st ^(7.4 μg/m^3^) to the 25^th ^(17.2 μg/m^3^), 50^th ^(33.8 μg/m^3^), 75^th ^(108.3 μg/m^3^), and 90^th ^(180.8 μg/m^3^) percentiles the adjusted estimated decreases in standardized birth weight were 0.04 (0.01), 0.07 (0.01), 0.12 (0.02) and 0.14 (0.03) z-score units respectively.^.^

There were significant interactions between black smoke and Townsend deprivation score on both birth weight and standardized birth weight for the whole pregnancy period (*P *= 0.003) and for trimesters 2 and 3 (*P *≤0.01. The estimated reduction in birth weight was 17 g (SE ± 9) for the 1st quintile (assumed to be most advantaged), 23 g (SE ± 10) for the 2nd, 42 g (SE ± 9) for the 3 rd, 27 g (SE ± 10) for the 4th quintile and 8 g (SE ± 11) for the 5th quintile (assumed to be the least advantaged) when exposure increased from the 1st percentile (7.4 g/m3) to the 25th percentile (17.2 g/m3) of whole pregnancy black smoke exposure. No other significant interactions were observed. In particular, there was no interaction between black smoke and time period on birth weight when considering the periods before (1962-77) and after (1978-92) the implementation of smokeless zones (*P *= 0.76).

For gestational age, the unadjusted estimate for the whole pregnancy was 0.02 weeks (95% CI: 0.01, 0.02) increase per 10 μg/m^3 ^increase in exposure based on the linear model. However, following adjustment for the covariates, the *P*-value rose to 0.67 with a 0.001 weeks (95% CI -0.003, 0.004) increase per 10 μg/m^3 ^increase in exposure.. Significant adjusted associations were seen for gestational age by trimester, corresponding to increases of 0.004 weeks per 10 μg/m^3 ^increase in black smoke for trimester 1, 0.003 weeks for trimester 2 and a decrease of 0.01 weeks for trimester 3 (Table 3).

## Discussion

In this study, using individual-level estimates of black smoke exposures during pregnancy, associations were seen between black smoke and birth weight, gestational age and birth weight standardized for gestational age. However, not all associations were significant and, regardless of whether significant, effect sizes tended to be fairly small, in particular for gestational age.

In contrast to most previous studies which have been ecological in design, with relatively crude measures of exposure, this study utilised individual-level exposure estimates on a large unselected birth cohort. Completeness of the PAMPER database for both the number of births and collected information for each birth is one of the evident strengths [19]. The study area covers a clearly defined conurbation with high quality records of land use, including historical records of industrial usage and numbers of residential and industrial chimneys, temperature and season, all of which was included within the exposure estimation modelling [26]. Although from a small geographical area, due to the long time period covered, the study included large variations in black smoke levels, ensuring an additional contribution to statistical power to that given by the large number of births. The black smoke recordings used in this study were collected routinely over the study period, although not all monitors were in place throughout the study period, creating likely geographical differences in uncertainties surrounding the estimated exposures. In addition, monitoring procedures naturally reflect the best practice at the time and, thus, while they are the best estimates available, their accuracy may have varied over time. It is also possible that those with shorter gestations will have more variability in exposure estimation for the whole of pregnancy and the last trimester. In contrast to other studies, we investigated non-linearity of the observed associations. In finding these, it remains to be seen if non-linearity is a true phenomenon, as a result of our data (as the fractional polynomial process is data driven), or due to residual confounding or due to the high level of correlation between black smoke and calendar year.

Residential mobility during pregnancy may be associated with exposure misclassification and therefore may introduce bias [28]. Whilst residential mobility data for the study cohort are not available, there is published, indirect, evidence of population stability for children and older women [21,22]. Further, only 9% of women in the north of England, in which this study is based, moved during pregnancy between 1985 and 2003, with a median moving distance of only 1.4 km [23]. Data on daily mobility were not available.

While many studies investigate influences on birth weight, or influences of birth weight on later health, many do not account for the impact of gestational age, a major determinant of birth weight. In this study, in addition to crude birth weight, a standardized measure was also used to give a better measure of fetal growth than birth weight alone. Different methods for gestational age assessment (based on the last normal menstrual period or early ultrasound measurements) throughout the study period may introduce bias in gestational age estimation over time. Gestational age in this study was made as objective and accurate as possible by accepting gestational age calculated from the recorded estimated date of delivery (i.e. last menstrual period based) for the majority of births, rather than by using gestational age as recorded in the neonatal notes or birth records. However, these methods are still prone to bias. Most home births in this study period were excluded due to a lack of outcome data. As the majority of home births were in the earlier decades of the study, they would have been associated with relatively high black smoke exposures compared to births in more recent years. However, due to the lack of information from birth ledgers, it was not possible to assess whether this introduced any bias.

Adjustment for year of birth in long-term studies of air pollution and birth outcomes is crucial. However, year of birth and black smoke exposure during pregnancy are highly correlated. While it is possible that adjustment for year of birth may reduce the strength of the association between black smoke and birth weight, it is also possible that residual confounding by year of birth or by other factors affecting birth weight and related to year of birth, for example increase in maternal overweight and improvement in quality of pre-natal care over the study period, may remain. There was a striking decrease in gestational age over the study period due to a number of reasons unrelated to air pollution. For example, the higher prevalence of obstetric interventions, including caesarean section, will account for much of the temporal decrease in gestational age.

The composition of black smoke is known to have varied over the study period [29], reflecting the diminishing importance of coal and increasing importance of vehicle traffic. From the late 1970s in Newcastle upon Tyne, there was a shift from domestic coal use for heating and cooking to smokeless fuel. However, there was no significant difference in the adjusted association between exposure to black smoke and birth weight between 1962-77 (the period before the implementation of smokeless zones in the area) and 1978-92. Although historical data from the north of England were used in this study, the levels as described still occur in many developing cities of the world. For example, the annual average PM_10 _concentrations in New Delhi were reported in 2005 to be above 150 μg/m^3^, and in Beijing above 100 μg/m^3 ^[30].

Two considerations are of relevance when comparing estimates of associations between black smoke or PM_10 _and birth outcomes: the nature of black smoke measurements means that their conversion factor to PM_10 _varies both over time and by composition [30]. For the geographical and temporal setting of this study, a study comparing black smoke and PM_10 _in the context of health studies concluded that daily average black smoke was a reasonable predictor of daily average PM_10 _[31]. In the present study, the non-linear relationship between black smoke and birth weight further complicated comparisons with previous studies that used linear models, therefore linear estimates were also reported as well as giving estimated effects at different percentiles of exposure to aid understanding of the functional forms used. The adjusted linear estimate of a 1.7 g (95% CI 0.9 to 2.4) decrease in birth weight per 10 μg/m^3 ^increase in average weekly black smoke exposure over the whole pregnancy period was lower than previous estimates (5, 8-10), but the estimates were more comparable in the non-linear models.

There was a significant interaction between community-level socio-economic status and black smoke on birth weight, with increasing estimated decreases in birth weight per 10 μg/m^3 ^with increasing socio-economic deprivation. Industrial processes in the UK cluster in areas of socio-economic deprivation, but attributing such unequal distribution to unequal pollution impact is complicated [32]. Other covariates that are known to affect birth weight and may be related to black smoke exposures, which we did not have access to, were ethnicity, maternal obesity and smoking. The ethnic minority of primary relevance in Newcastle upon Tyne are from the Indian subcontinent (4% in 1991), therefore the potential for confounding by ethnicity is likely to be small.

A pooled analysis showed that passive smoking reduced birth weight on average by 31 g, a meta-analysis reported a 28 g reduction [33,34]. The estimated effect of black smoke exposure on birth weight in this study is, therefore, of a similar order of magnitude as the effect of passive smoking, but is, on the other hand, much weaker than the effect of active smoking [35]. Smoking levels in women in the UK never exceeded 45%. For the 1990s we know that smoking was more prevalent in lower socio-economic groups, whereas in the 1960s the prevalence of smoking was high across all social strata. During the 1970s and 80s smoking rates fell sharply in the non-manual occupational groups, leading to the still widening gap between socio-economic groups that exist today. Adjusting for neighbourhood deprivation controls well for smoking to some extent, at least since the 1980s [36].

Although the association between black smoke and gestational age was not statistically significant overall, it was for each trimester. Given the inconsistent directions between the trimester associations, it is likely that they cancelled out an association for the whole of pregnancy, although the estimated effect sizes were very small. Given the associations between black smoke and both birth weight and standardized birth weight were stronger, it is more likely that the observed associations point towards intrauterine growth restriction as a potential causal pathway for the black smoke effect.

Slama et al. [17] summarised in detail the potential mechanisms for the association of air pollution on intrauterine growth restriction. These include alterations of umbilical and utero- placental blood flow, and deterioration in the transport of glucose and oxygen to the fetus, all of which can influence fetal growth. Air pollution has been previously associated with increased risks of pre-eclampsia and pregnancy induced hypertension [37]. Data on these outcomes were not available to this study.

## Conclusions

The findings of this study push forward the hypothesis that the association between black smoke and birth weight may be mediated through intrauterine growth restriction. The estimated effects of black smoke on birth weight were found to be in the same order of magnitude as those for passive smoking exposure. Findings from this study contribute to the growing evidence regarding the adverse effect of ambient air pollution on birth weight. Future reviews of air quality standards should include assessments of fetal growth.

## Abbreviations

CI: Confidence interval; PAMPER: Particulate matter and perinatal events research; PM_10_: Particulate matter with an aerodynamic diameter of 10 micron or less; SD: Standard deviation; SE: Standard error; UK: United kingdom

## Competing interests

The authors declare that they have no competing interests.

## Authors' contributions

The PAMPER study was conceived by LP and TPM, who designed this study with MSP, SVG, JR and MC. Outcome data collection was led by SVG and geographical data collection by MC. Analysis was done by RG, under the supervision of MSP and SR. MSP wrote this manuscript with assistance from all other authors. All authors read and approved the final manuscript.
